# Supplementary figures and images for: A spatio-temporal assessment of simian/human immunodeficiency virus (SHIV) evolution reveals a highly dynamic process within the host
Source: PLoS Pathog. 2017 May 25;13(5):e1006358. doi: 10.1371/journal.ppat.1006358 (PMC5444849; doi:10.1371/journal.ppat.1006358)

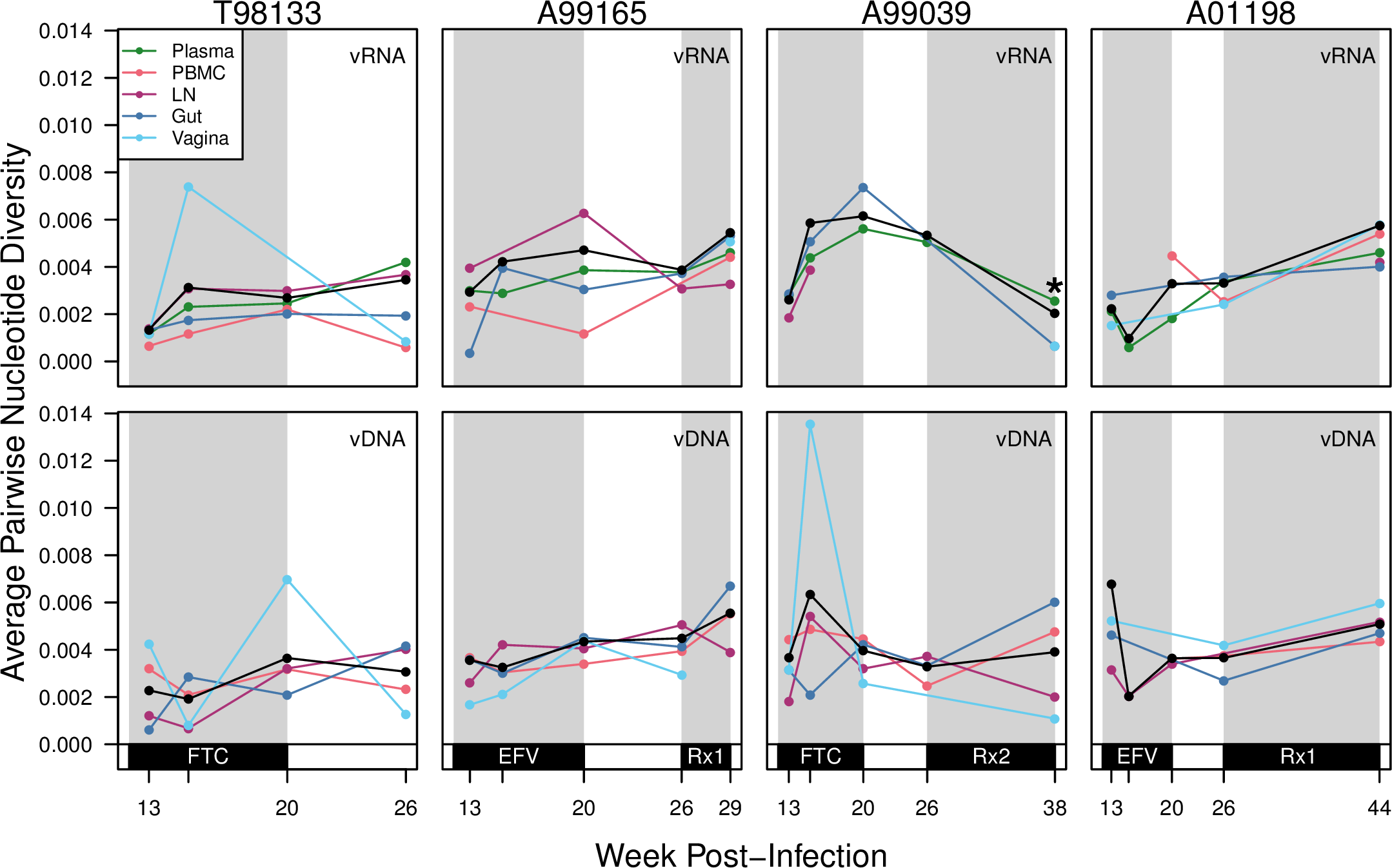

Supplement: S1 Fig — The average pairwise diversity (π) increases over time in both the vRNA (top row) and vDNA (bottom row) in each macaque (shown in the columns). Each colored line marks a different compartment with the diversity of all compartments combined shown in black. The asterisk marks where diversity decreases at the final sampling point for A99039, and corresponds to viral suppression (see Fig 1). Grey shading indicates monotherapy or combination therapy, as indicated below the x-axis. Rx1 is treatment FTC+TFV+EFV and Rx2 is the treatment TFV+L870812+DRV/r. (TIF) [file ppat.1006358.s003.tif]

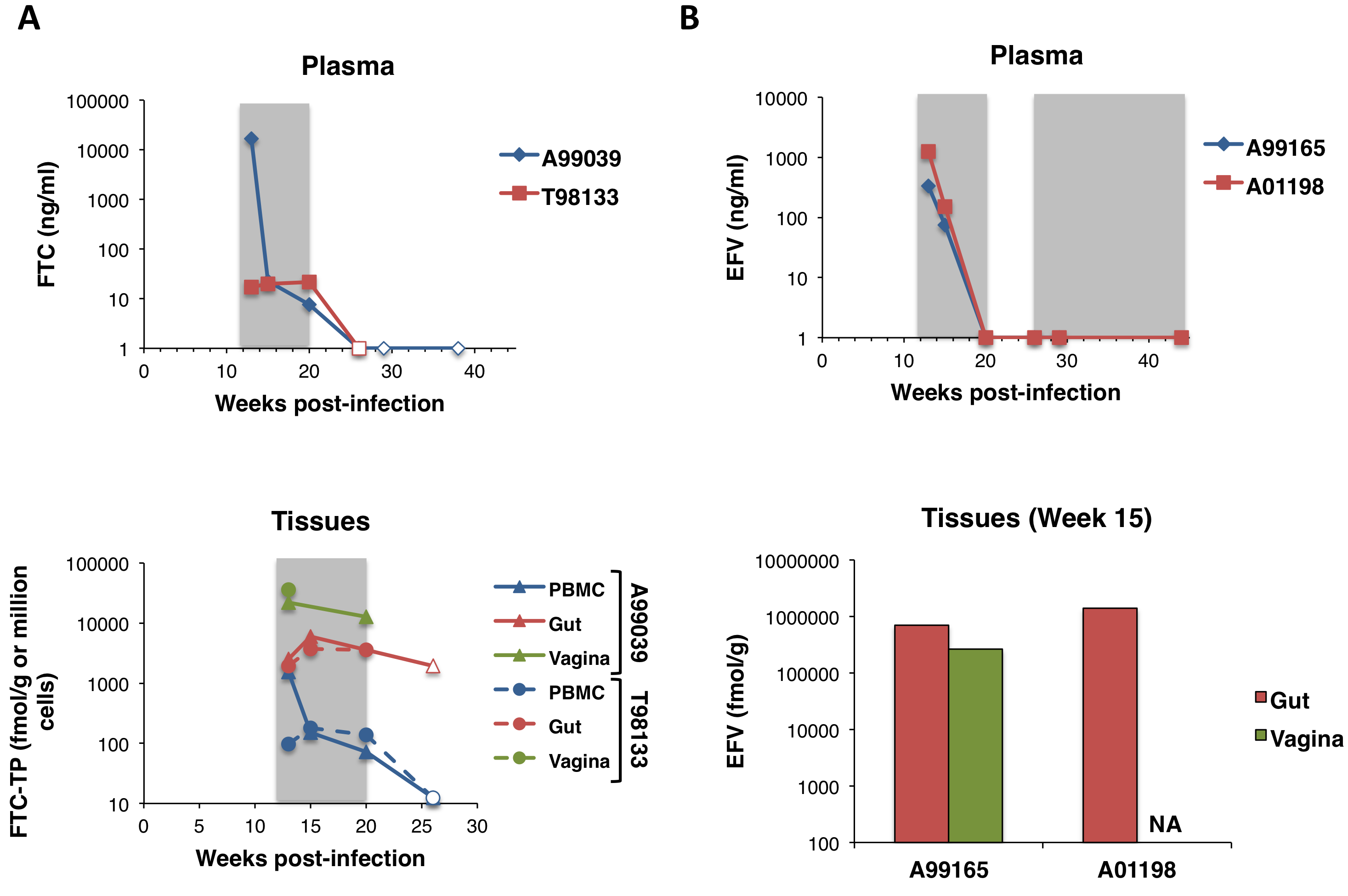

Supplement: S2 Fig — FTC and FTC-TP (A) and EFV (B) concentrations were measured in limited plasma (top) and PBMC and mucosal biopsy samples (bottom) taken from all animals at multiple time points. Grey shading indicates monotherapy (weeks 12–20). Open symbols represent measurements that were below the lower level of quantitation. NA indicates sample was not available. Unfortunately LN tissues were not available for drug measurements. (TIFF) [file ppat.1006358.s004.tiff]

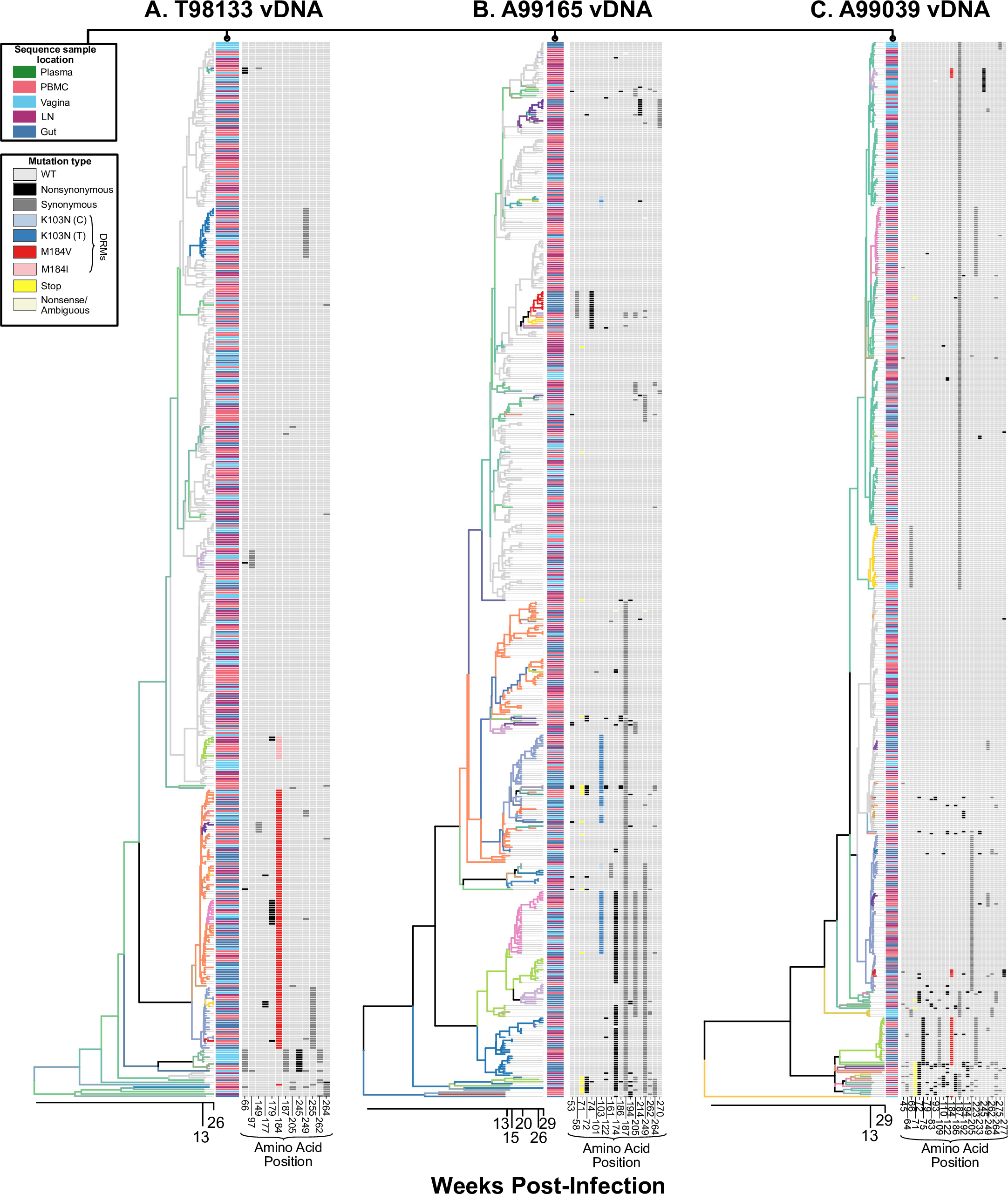

Supplement: S3 Fig — Branch lengths indicate sequence sampling time from vDNA of macaques T98133 (A), A99165 (B), and A99039 (C). Branches are colored to match the corresponding plots of Figs 3, 4 and 5(A) and 5(B) for T98133, A99165 and A99039, respectively. Sampling location is indicated for each sequence, and the identity of all mutations at frequency >1% is shown to the right. Colors indicating mutation type (synonymous, nonsynonymous, DRM, stop or nonsense/missense) are shown in the legend. (TIF) [file ppat.1006358.s005.tif]

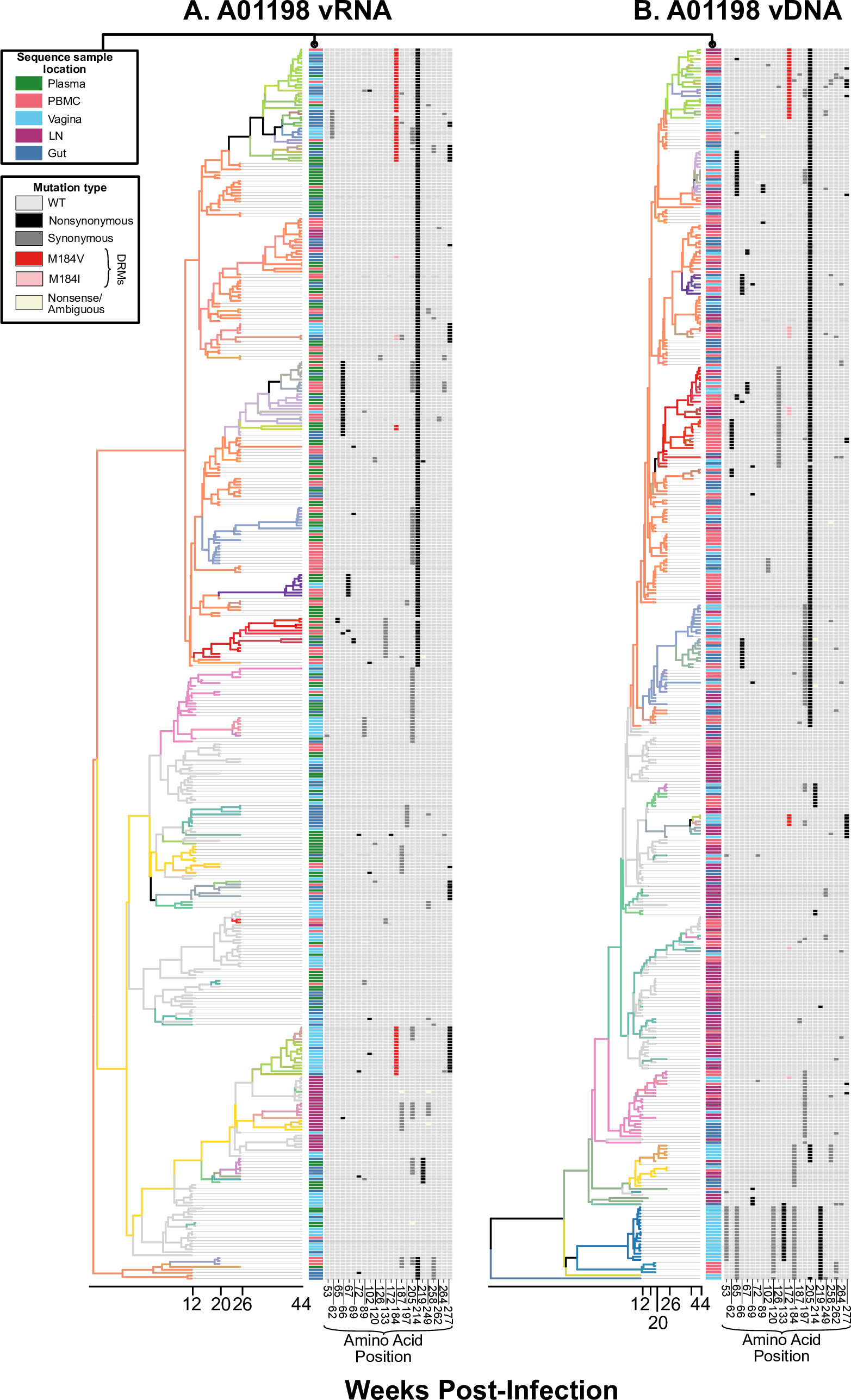

Supplement: S4 Fig — Branch lengths indicate sequence sampling time from the vRNA (A) and vDNA (B) of macaque A01198. Sampling location is indicated for each sequence, and the identity of all mutations at frequency >1% is shown to the right. Colors indicating mutation type (synonymous, nonsynonymous, DRM, stop or nonsense/missense) are shown in the legend. (TIF) [file ppat.1006358.s006.tif]

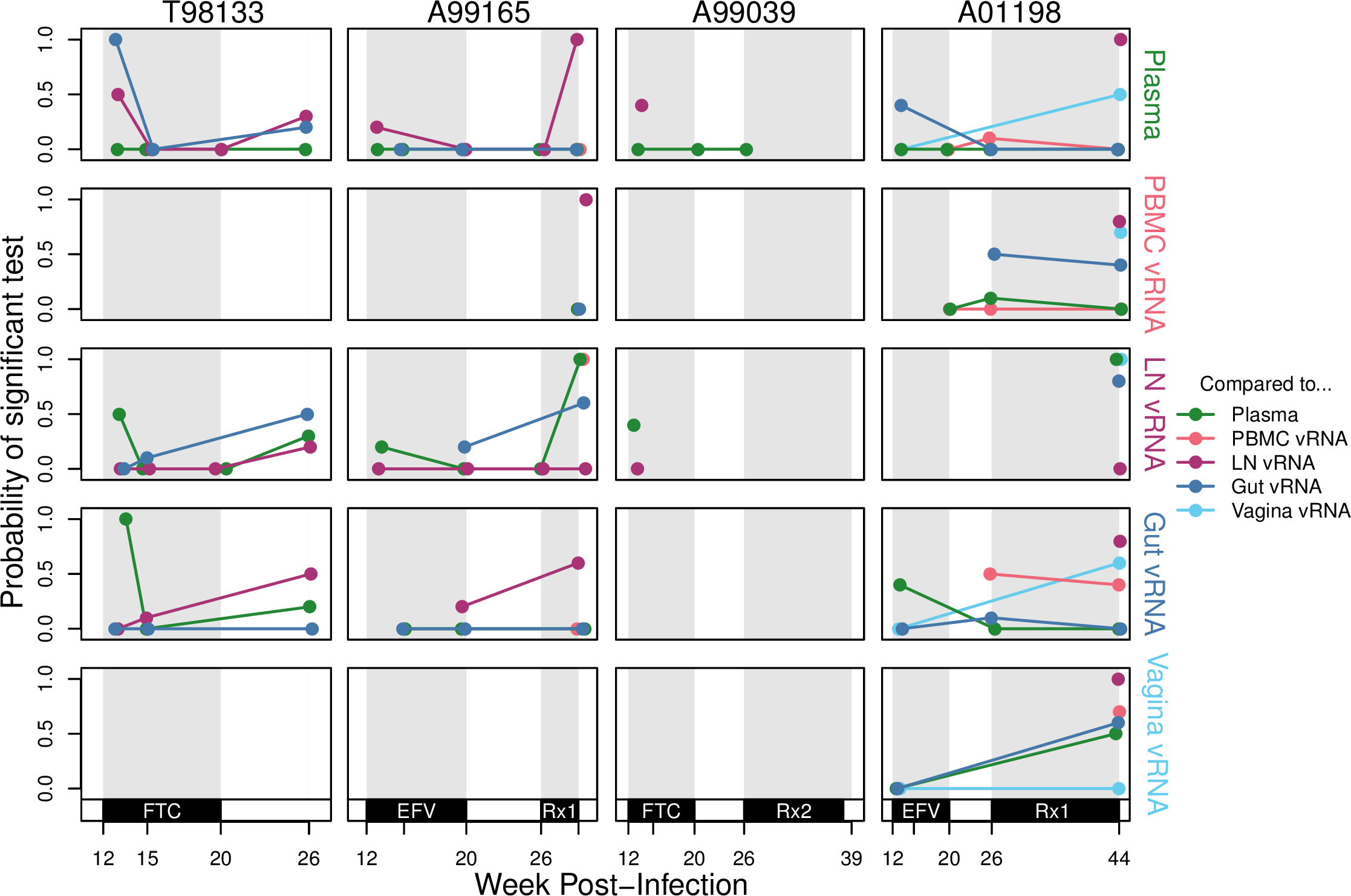

Supplement: S5 Fig — The y-axis indicates the proportion of Slatkin-Maddison tests significant at the 5% significance level when subsampled to 10 sequences per compartment 100 times, but the figure caption is otherwise shared with Fig 6. (TIF) [file ppat.1006358.s007.tif]

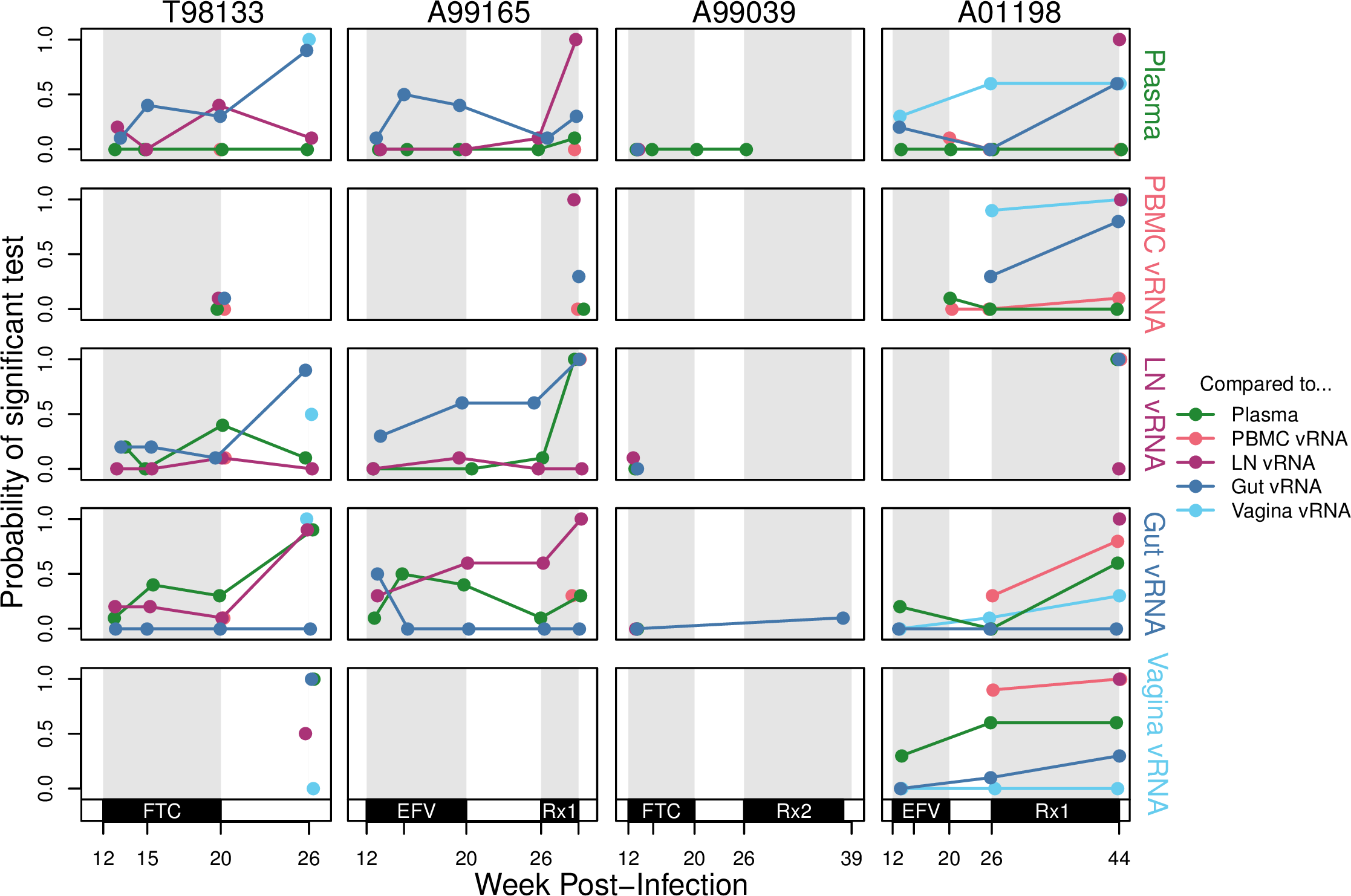

Supplement: S6 Fig — The y-axis indicates the proportion of AMOVA tests significant at the 5% significance level when subsampled to 10 sequences per compartment 1000 times, but the figure caption is otherwise shared with Fig 6. (TIF) [file ppat.1006358.s008.tif]

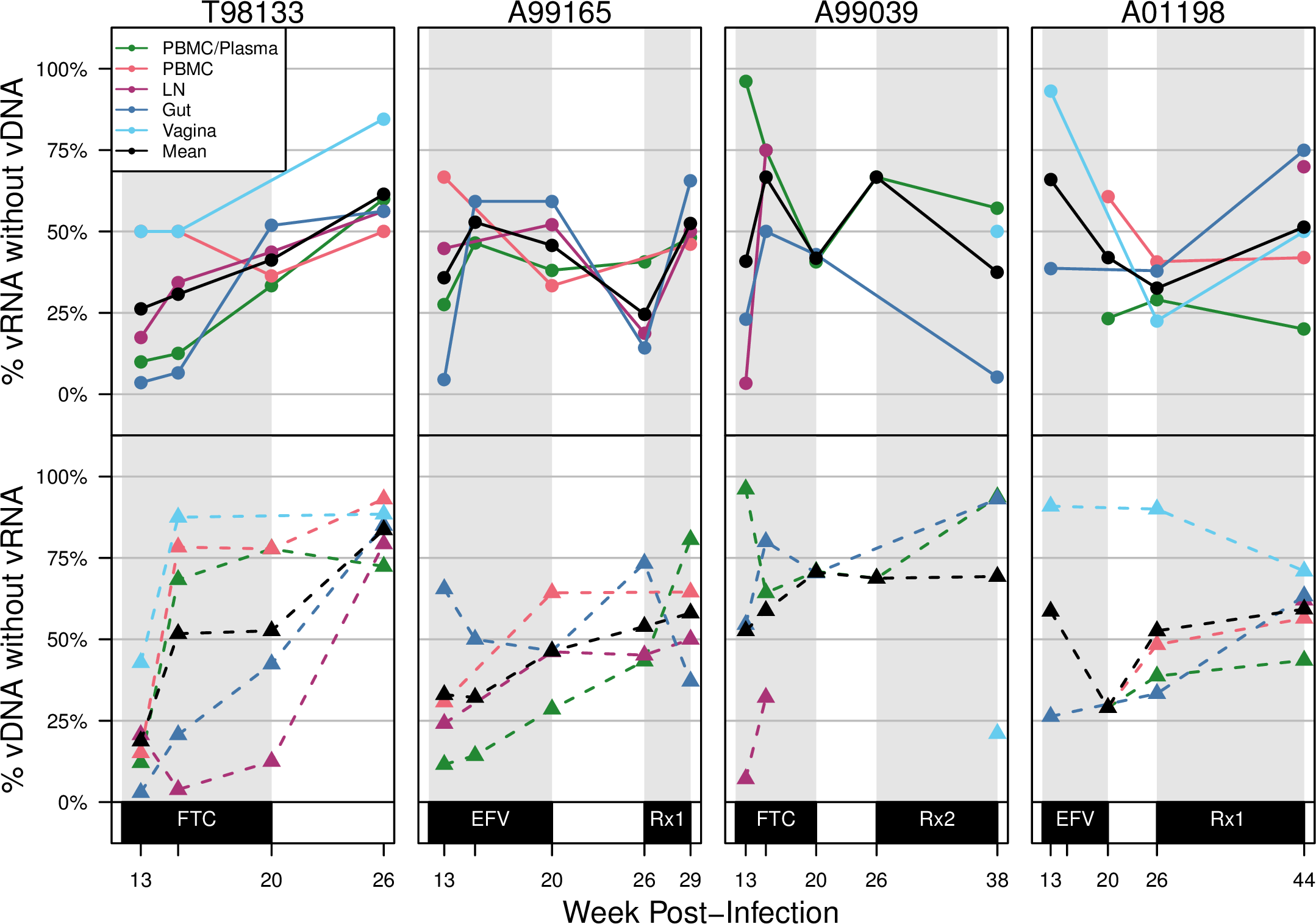

Supplement: S7 Fig — For a given compartment, the percentage of vRNA genotypes without corresponding vDNA genotypes (top) as well as the the percentage of vDNA genotypes without corresponding vRNA genotypes (bottom) is plotted at each time point, in which a genotype was present in both vRNA and vDNA for a given compartment, macaque and time point at a frequency greater than 3 sequences. Plasma vRNA is compared to PBMC vDNA (green), and all other samples are compared within their sampled compartment (lymph node in purple, vagina in light blue, gut in dark blue and PBMC vRNA verus vDNA in pink). The time point means for both vDNA without vRNA and vRNA without vDNA are shown in black and show overall trends across compartments. Time points that were within a week of each other were considered close enough to compare. Grey shading indicates monotherapy or combination therapy, as indicated below the x-axis. Rx1 is treatment FTC+TFV+EFV and Rx2 is the treatment TFV+L870812+DRV/r. (TIF) [file ppat.1006358.s009.tif]

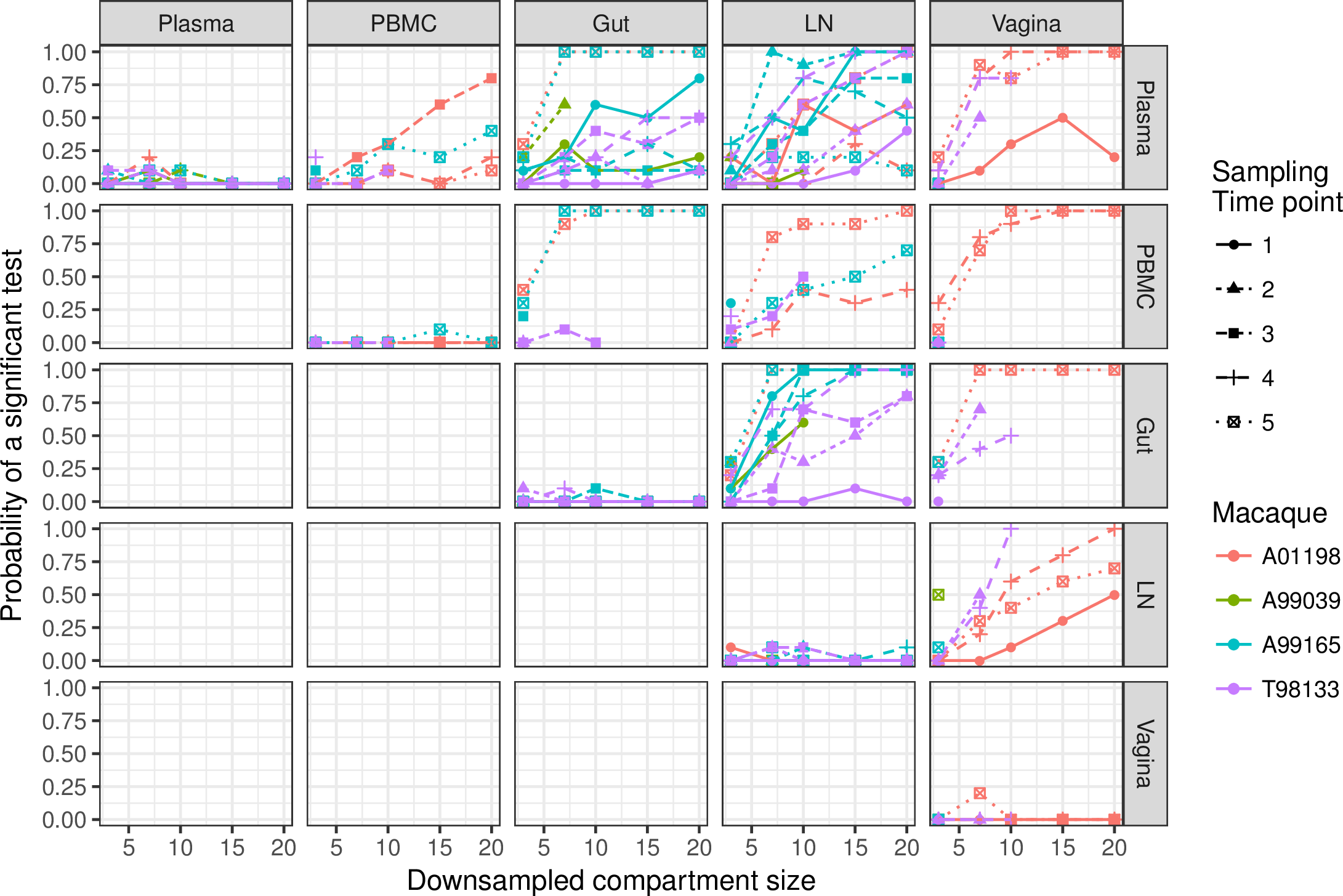

Supplement: S8 Fig — For each pairwise compartmental comparison, the proportion of 100 subsampled KST tests resulting in a permutation test p-value ≤ 0.05 is plotted versus the down sampling number separately by macaque and paired time points (i.e., time points within ±1 week). Sequences sampled from different macaques are shown in different colors, and compartments sampled from different weeks are represented with different symbols. Lines connect tests from the same macaque and the same week at different levels of subsampling. Because subsampling is done without replacement, lines extend only as far as the minimum of the sample size of the two compartments being compared. The time point represents the relative time of sampling, with time point 1 representing weeks 12 or 13, time point 2 representing weeks 15 or 16, time point 3 representing weeks 20 or 21, time point 4 representing weeks 26 or 27, and time point 5 representing week 29+. (TIF) [file ppat.1006358.s010.tif]

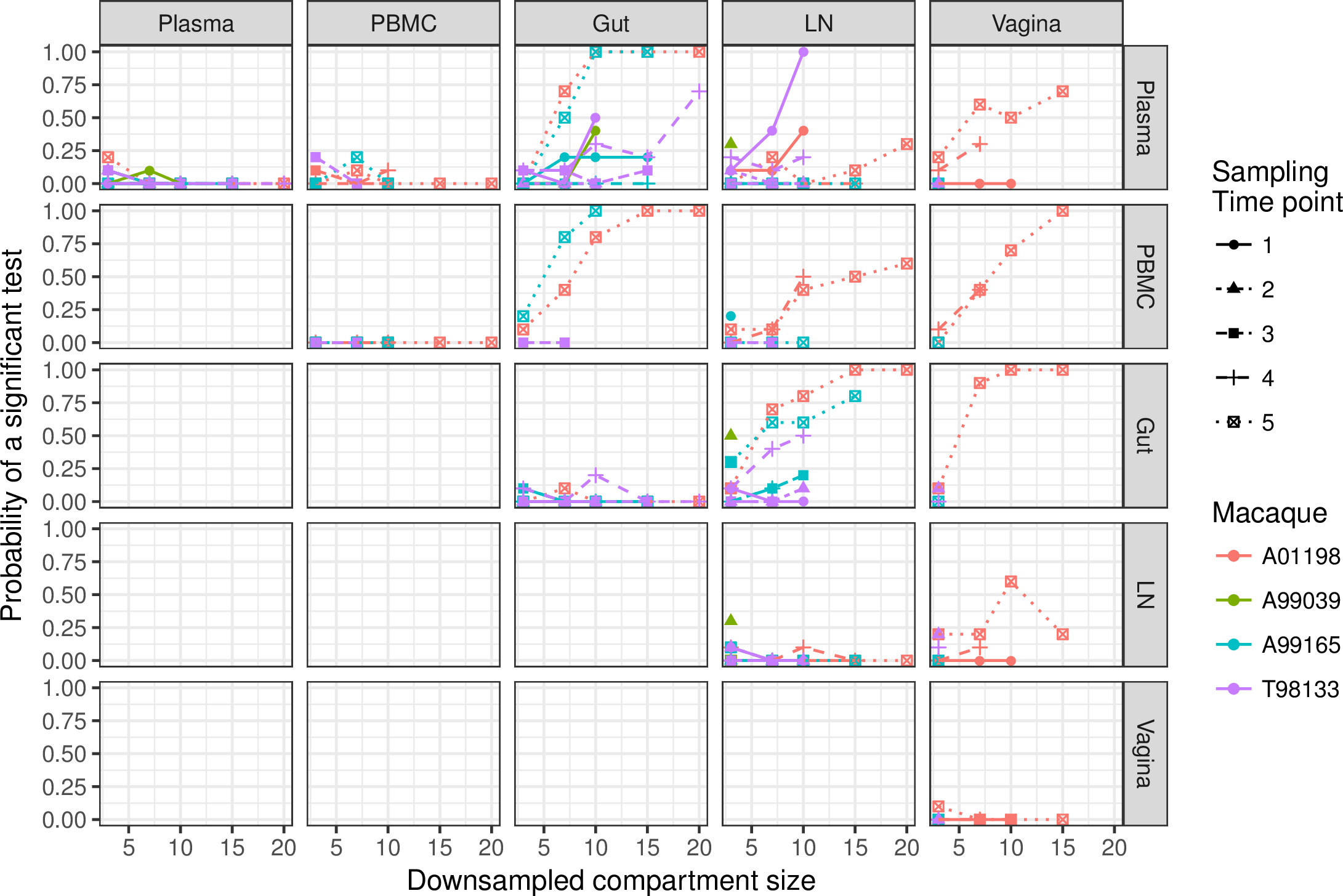

Supplement: S9 Fig — The y-axis represents the proportion of 100 subsampled Slatkin-Maddison tests resulting in a permutation test p-value ≤ 0.05, but the caption is otherwise shared with S6 Fig. Slatkin-Maddison tests are done using only unique sequences, as described in the Materials and Methods. (TIF) [file ppat.1006358.s011.tif]

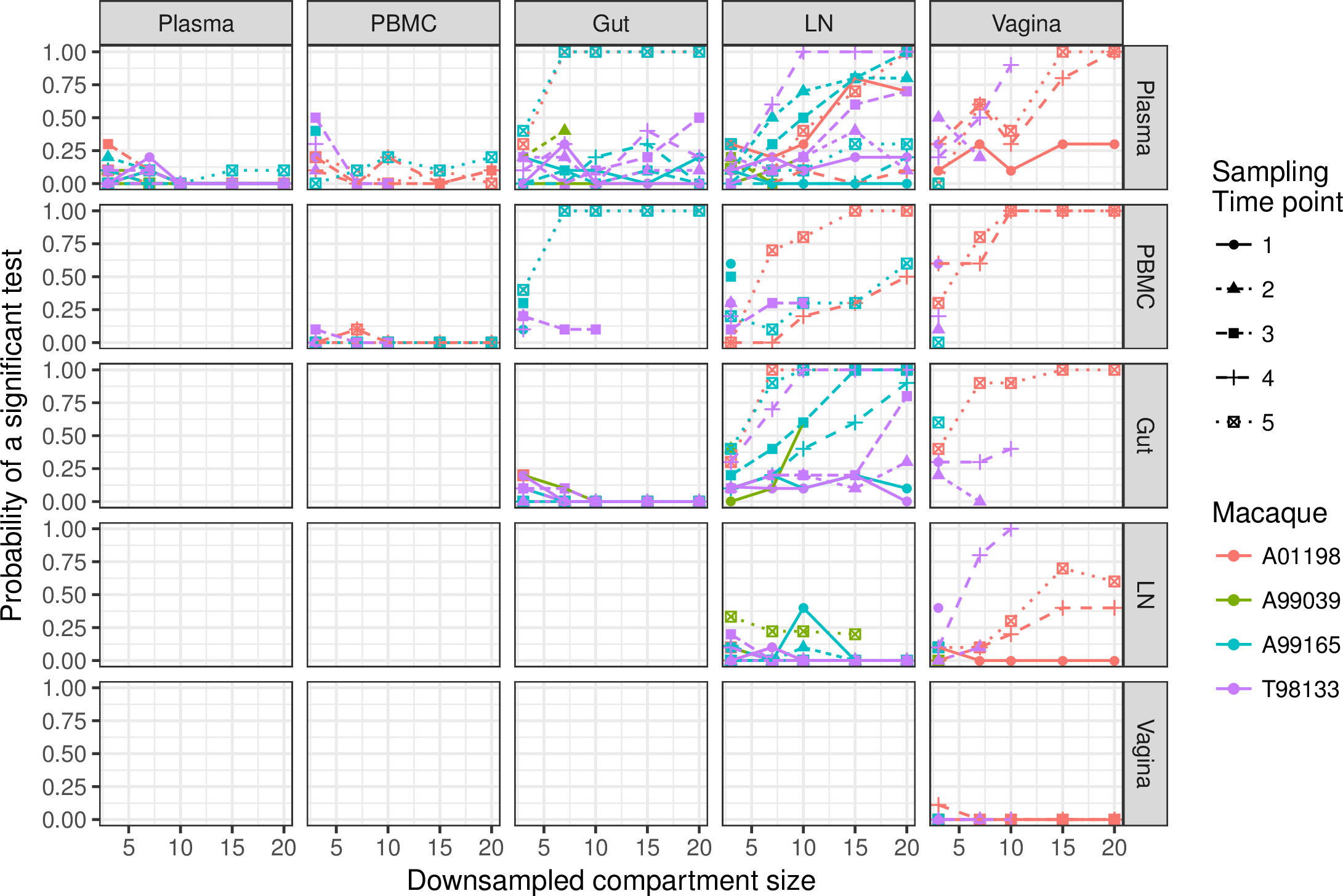

Supplement: S10 Fig — The y-axis represents the proportion of 100 subsampled AMOVA tests resulting in a permutation test p-value ≤ 0.05, but the caption is otherwise shared with S6 Fig. (TIF) [file ppat.1006358.s012.tif]
